# Which frequency is better for pediatric shock wave lithotripsy? Low intermediate or high: A systematic review and meta-analysis

**DOI:** 10.3389/fsurg.2023.1063159

**Published:** 2023-03-15

**Authors:** Kaiwen Xiao, Liang Zhou, Shiyu Zhu, Lede Lin, Xingpeng Di, Hong Li

**Affiliations:** Department of Urology/Institute of Urology, West China School of Medicine, West China Hospital, Sichuan University, Chengdu, China

**Keywords:** ESWL, frequency, pediatric, urolithiasis, meta-analysis

## Abstract

**Background:**

To explore the optimal frequency for pediatric extracorporeal shock wave lithotripsy (ESWL) in the treatment of upper urinary stones.

**Methods:**

A systematic literature search was undertaken using PubMed, Embase, Web of Science and Cochrane Central Register of Controlled Trials databases to identify eligible studies published before January 2023. Primary outcomes were perioperative efficacy parameters, including ESWL time, anesthesia time for ESWL sessions, success rates after each session, additional interventions needed, and treatment sessions per patient. Secondary outcomes were postoperative complications and efficiency quotient.

**Results:**

Four controlled studies involving 263 pediatric patients were enrolled in our meta-analysis. In the comparison between the low-frequency and intermediate-frequency groups, we observed no significant difference as regards anesthesia time for ESWL session (WMD = −4.98, 95% CI −21.55∼11.58, *p* = 0.56), success rates after ESWL sessions (first session: OR = 0.02 95%CI −0.12∼0.17, *p* = 0.74; second session: OR = 1.04 95%CI 0.56∼1.90, *p* = 0.91; third session: OR = 1.62 95%CI 0.73∼3.60, *p* = 0.24), treatment sessions needed (WMD = 0.08 95%CI −0.21∼0.36, *p* = 0.60), additional interventions after ESWL (OR=0.99 95%CI 0.40∼2.47, *p* = 0.99) and rates of Clavien grade 2 complications (OR = 0.92 95%CI 0.18∼4.69, *p* = 0.92). However, the intermediate-frequency group may exhibit potential benefits in Clavien grade 1 complications. In the comparison between intermediate-frequency and high-frequency, the eligible studies exhibited higher success rates in the intermediate-frequency group after the first session, the second session and the third session. More sessions may be required in the high-frequency group. With respect to other perioperative, postoperative parameters and major complications, the results were similar.

**Conclusions:**

Intermediate-frequency and low-frequency had similar success rates and seemed to be the optimal frequency for pediatric ESWL. Nevertheless, future large-volume, well-designed RCTs are awaited to confirm and update the findings of this analysis.

**Systematic Review Registration:**

https://www.crd.york.ac.uk/prospero/, identifier: CRD42022333646.

## Introduction

Extracorporeal shock wave lithotripsy (ESWL) has been in service since the early 1980s (1); since then, it has rapidly become a widely accepted treatment method with satisfactory safety as well as efficacy for both renal stones and ureteral stones. ESWL was first used successfully by Newman in 1986 for the treatment of pediatric urolithiasis (2), and this management of urolithiasis in children has received increasing attention from pediatric urologists. It is accepted that pediatric patients have an increased clearance rate of stones compared to adults (3). Following this, it is often regarded as the first-line treatment approach for most urinary stone diseases in the pediatric population in the current urology field (4–6).

Previous studies have reported that there are some variables that could influence the efficiency of ESWL on stone fragmentation, involving stone location, stone size and composition, shock wave number, design of lithotripter, output energy and frequency (7, 8). Therein, stone disintegration is influenced by the rate of shock wave (SW), which has been demonstrated by both *in vitro* and animal studies. However, some conflicting results exist, and several studies have reported that decreasing the frequency to less than 120 SW/minute may improve stone fragmentation (9).

Although several comparative studies of the frequency of pediatric shock wave lithotripsy have been published up to the present, no systematic appraisal has been performed. Hence, this systematic review and meta-analysis of controlled studies was conducted to determine the appropriate frequency from low frequency, intermediate frequency, and high frequency for upper urinary stones in pediatric patients. Terms of comparison included anesthesia time, success rates, treatment sessions, additional procedures required after ESWL sessions, complications and efficiency quotient.

## Methods

### Selection criteria for the included studies

Published studies that conformed to the following criteria were involved: (1) Study designed to assess the effect of different SWL frequencies to treat renal stones in the pediatric population. (2) full papers reporting on at least one of the two primary outcomes of efficacy (mainly evaluated by stone-free rate or success rate) and safety from two or more groups. (4) Standard indications for ESWL in clinical treatment. (5) End-point outcome parameters also included the complication rate. The current systematic review was performed following the PRISMA statement (10) and was registered in PROSPERO (No. CRD42022333646).

### Search strategy

Electronic databases involving PubMed, Embase, Web of Science and the Cochrane Central Register of Controlled Trials were systematically retrieved to identify relevant controlled studies. The time frame spanned between January 1, 2000, and January 12, 2022. The retrieval in the current study is not restricted by region or language. Search strings were applied as follows: shock wave lithotripsy, pediatric, rate and frequency.

### Data extraction

Titles and abstracts were applied in the advanced literature search to retrieve studies, and only full-text articles were finally selected. Then, the details were further reviewed to evaluate their eligibility for the current review. We tried to contract the corresponding author for more specific information if some crucial information was not available in the published paper. Two investigators extracted the data independently by using pre-set tables. Baseline characteristics of patients involving the first author’s name, publication year, interventions, age, gender, BMI, stone site, stone size, stone location, total shock pulses, and maximum power applied. Treatment outcome parameters included ESWL time, anesthesia time for ESWL sessions, success rates after each session, additional interventions required, treatment sessions per patient, postoperative complications and efficiency quotient. Other study-related data, including the definition of success, time of follow-up, and frequency of ESWL administration, were also filed.

**Figure 1:**
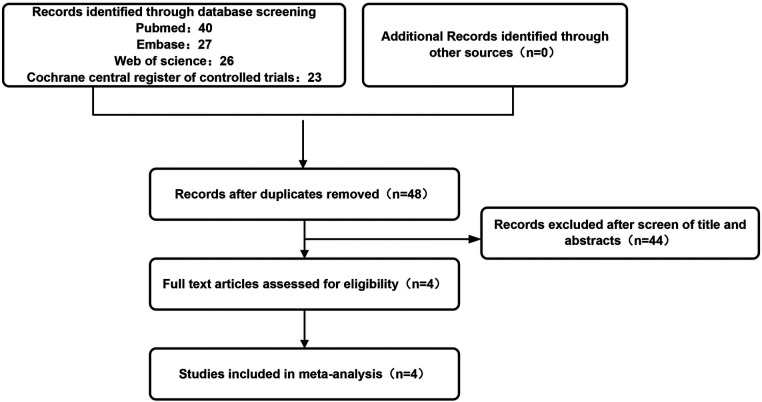
The flow chart showing the retrieval procedures.

A total of 263 pediatric patients from four independent studies (11–14) conformed to the predetermined inclusion criteria and were involved in our present review. Therein, three studies compared the effects of ESWL at low frequency vs. intermediate frequency. Two RCTs compared the effects of ESWL at intermediate frequency vs. high frequency. The characteristics of patients included in different studies are summarized in Table 1. The physical parameters of the utilized extracorporeal shock wave lithotripsy devices are exhibited in Table 2. The literature filtration method is illustrated in Figure 1. All four studies were included in the pooled meta-analyses, containing three RCTs and one retrospective study. The quality evaluations of RCTs and non-RCTs are exhibited in Tables 3, 4, respectively. For RCTs, blinding of the surgeons was impossible with regard to the nature of the clinical intervention; hence, if the blinding of the patients and the outcome estimators were described in the methodology section, the study was deemed blinded. For non-RCTs, the only eligible study was evaluated as high quality.

**Table 1:** Baseline characteristic of included studies.

Location												
	Interventions (SWs/min)	No. of patients	Male / Female	Age	BMI (kg/m^2^)	Side (L/R)	Stone size (mm)	Pelvis	Mid or upper calyx	Lower calyx	Lower calyx stones that include multiple calyx	Multiple calyx
Kaygisiz 2021	60	38	15/23	4 (1–16)	16.3 (10.4–23.6)	20/18	9 (5–19)	10	17	8	3	5
	90	33	19/14	5 (1–16)	16.3 (12.8–23.1)	18/15	8.5 (5–19)	8	14	6	5	7
Tuncer 2021	60	25	13/12	3 (1–17)	18 (13.6–25.9)	14/11	10.29 ± 3.47	20	5	0	0	
	90	25	14/11	4 (1–11)	15.9 (11.7–28.1)	16/9	9.98 ± 2.50	21	4	0	0	
	120	25	9/16	3.5 (0–14)	15.9 (11.9–28.3)	11/14	11.23 ± 3.65	22	3	0	0	
Kaygisiz 2018	60	30	19/11	3 (1–12)	16.2 (8.33–19.55)	17/13	9.57 ± 3.19	9	3	5	13	13
	90	27	15/12	6 (1–12)	16.04 (13.19–21.30)	14/13	10.41 ± 3.22	9	4	9	12	5
Salem 2014	80	30	20/10	5.35 ± 3.67 (3-14)	22.7 ± 2.5	18/12	15.02 ± 2.47	15	6	9	0	0
	120	30	18/12	6.67 ± 2.94 (3-13)	23.4 ± 3.1	17/13	13.85 ± 3.42	18	7	5	0	0

**Table 2:** Physical parameters of the utilized extracorporeal shock wave lithotripsy devices.

Studies	Interventions (SWs/min)	System	Manufacturers	Total shock pulses median (min-max)	Energy output
Kaygisiz 2021	60	Ungated electrohydraulic lithotripter	Elmed Medical Systems, Ankara, Turkey	4,000 (2,000–6,000)	19 (18–19) KVs
	90			2,000 (1,800–6,500)	19 (18–19) KVs
Tuncer 2021	60	Electromagnetic shock wave generating system	Dornier Compact Sigma; Med Tech, Munich, Germany	2,800 (1,200–7,800)	0.029 J/SW
	90			3,100 (1,100–6,500)	0.030 J/SW
	120			3,400 (1,500–7,060)	0.031 J/SW KVs
Kaygisiz 2018	60	Ungated electrohydraulic lithotripter	Elmed Medical Systems, Ankara, Turkey	2,000 (1,600–5,800)	17 (17–18) Kvs
	90			3,600 (1,600–6,700)	17 (16–18) KVs
Salem 2014	80	Dornier Lithotripter S	Dornier Medical Systems, Kennesaw, Georgia	NA	14–24 KVs
	120			NA	14–25 KVs

**Table 3:** Risk of bias for RCTs.

Studies	Random sequence generation (selection bias)	Allocation concealment (selection bias)	Blinding of participants and personnel (performance bias)	Blinding of outcome assessment (detection bias)	Incomplete outcome data (attrition bias)	Selective reporting (reporting bias)	Other bias
Kaygisiz 2021	low risk	low risk	unclear risk	unclear risk	low risk	low risk	unclear risk
Tuncer 2021	low risk	unclear risk	unclear risk	unclear risk	low risk	low risk	unclear risk
Salem 2014	low risk	unclear risk	low risk	unclear risk	low risk	low risk	low risk

**Table 4:** Newcastle-Ottawa scale for quality assessment of controlled trial.

Study	Selection				Comparability	Outcome			Total score
	Representativeness of the exposed cohort	Selection of the nonexposed cohort	Ascertainment of exposure	Outcome of interest was not present at start of study	Based on the design or analysis	Assessment of outcome	Follow-up long enough for outcomes to occur	Adequacy of follow-up of cohorts	
Kaygisiz 2018	1	1	1	1	2	1	1	1	9

Tuncer et al. compared 90 SWs/min and 120 SWs/min shock wave output frequency in disintegrating pediatric kidney stones, while Salem et al. compared 80 SWs/min with 120 SWs/min. To facilitate inclusion analysis, we inclined to determine 80 SWs/min and 90 SWs/min as intermediate frequency, the 120 SWs/min as high frequency, and the 60 SWs/min as low frequency.

### Risk of bias assessment

We assessed the included studies based on different levels of evidence (LE) according to the grade of evidence (Oxford Centre for Evidence-based Medicine Website) (15). Two of the authors independently evaluated the risk of bias of the included studies. The “Risk of bias” tool recommended by the Cochrane Handbook of Reviews of Effectiveness of Interventions was applied in the assessment of eligible RCTs. For non-RCTs, the Newcastle‒Ottawa Scale (NOS) (the score ranges from 0 to 9) was used, and when a study had a score more than 7, it was identified as qualified enough to be involved in the final analysis (16). If agreement could not be reached by the two authors, an independent third arbiter was consulted.

### Statistics

The current study was conducted using Review Manager (version 5.4). Continuous variables are presented as the mean ± standard deviation. When the standard deviation of various comparative points was not available in an involved study, the following formula was applied: a = min, b = max, m = median, mean = (a + 2m + b)/4, n ≤ 25 or mean = m, *n* < 25; SD = (b-a)/4, 15 < *n* ≤ 70 (17). For the dichotomous variables, odds ratios (ORs) and 95% confidence intervals (CIs) were used.

### Heterogeneity test inconsistency assessment

The I2 statistic was applied to assess the inconsistency among the studies. When I2 < 25%, 25%≤I2 > 50% or I2 > 50%, low, moderate, and high heterogeneity was considered, respectively. If *p* > 0.1 or I2 ≤ 50%, the consistency of the included studies was relatively good, or else, a random-effects model was utilized because of the high heterogeneity among the investigations.

### Subgroup and sensitivity analyses

The potential defects could not be tested by performing subgroup analyses or deleting a single study because only four studies qualified enough for the final analyses.

## Results

Overall, no significant effects were acquired in the meta-analyses of baseline parameters, except for the total shock pulses between intermediate frequency and high frequency (total shock pulses: WMD = −0.15, 95% CI −0.22∼-0.08, *p* < 0.01).

### Anesthesia time for the ESWL session

In the comparison of the anesthesia time for SWL session between low-frequency and intermediate-frequency, no statistically significant difference was detected (WMD = −4.98, 95% CI −21.55∼11.58, *p* = 0.56, 93 patients in the low-frequency group, 85 patients in the intermediate-frequency group). Both Tuncer et al. and Salem et al. reported the comparison of anesthesia time for SWL session between intermediate frequency and high frequency. However, Salem et al. showed a significantly lower mean anesthesia time in the high frequency group, while Tuncer et al. revealed no statistically significant difference regarding this parameter.

### Success rates after ESWL sessions

**Figure 2:**
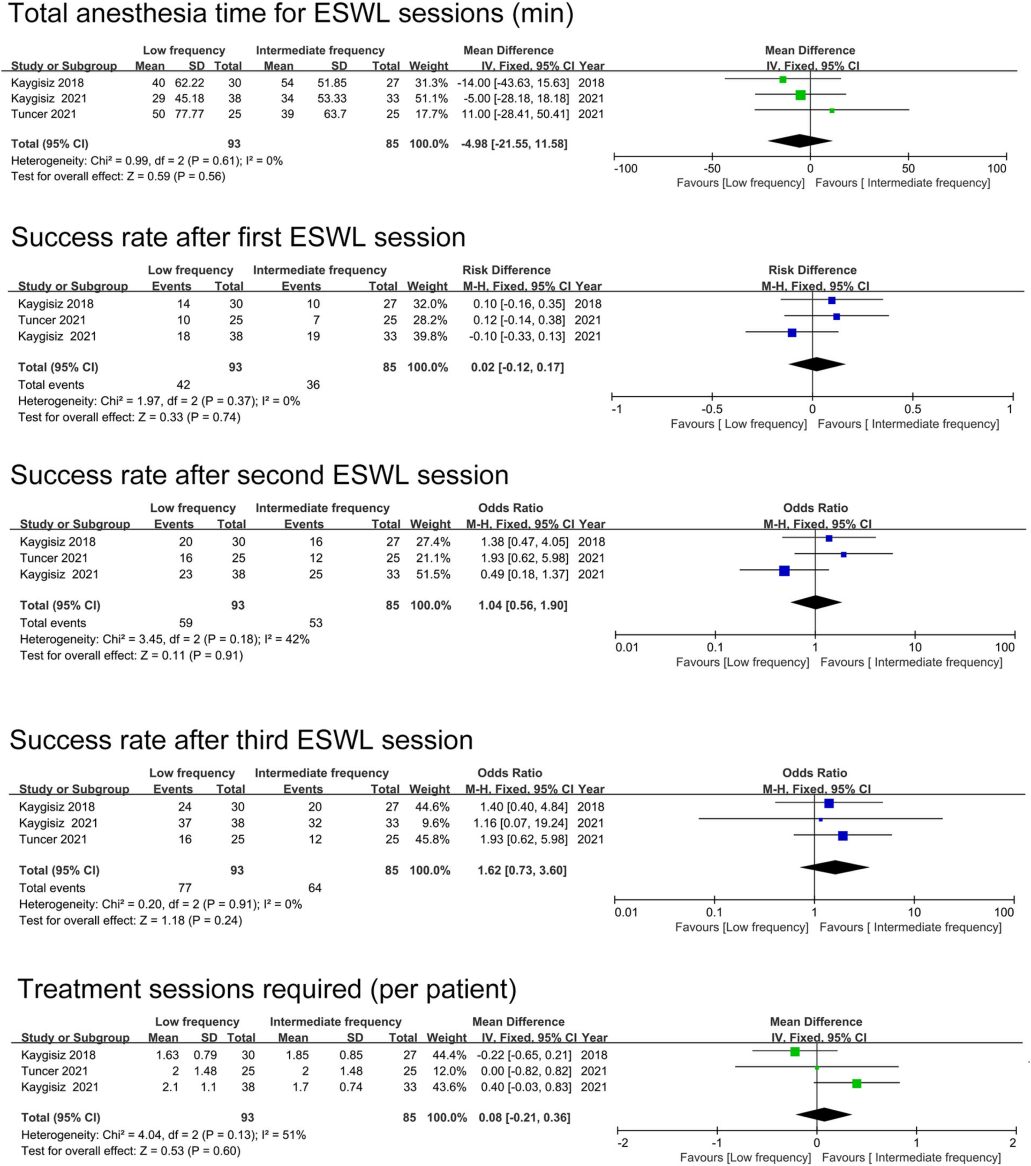
The pooled analyses of anesthesia time for ESWL sessions, success rate after sessions and treatment sessions required.

We compared the success rates after ESWL sessions for low frequency vs. intermediate frequency. Three studies were finally involved in the meta-analyses, and the pooled results exhibited no statistically significant difference concerning the first session, the second session or the third session (first session: OR = 0.02 95%CI −0.12∼0.17, *p* = 0.74; second session: OR = 1.04 95%CI 0.56∼1.90, *p* = 0.91; third session: OR = 1.62 95%CI 0.73∼3.60, *p* = 0.24) (Figure 2). Two RCTs were enrolled in the comparison of success rates after ESWL sessions between the intermediate frequency and high frequency. Significantly higher success rates were found in the intermediate frequency group after the first session, the second session and the third session in both studies.

### Treatment sessions needed

Three studies reported the number of sessions needed per patient in the low-frequency group vs. the intermediate-frequency group (Figure 2), and no significant distinction was observed (WMD = 0.08 95%CI −0.21∼0.36, *p* = 0.60). In the high-frequency group, Both Salem et al. and Tuncer et al. found significantly more sessions needed to become stone free compared with the intermediate-frequency group.

### Additional interventions after ESWL

**Figure 3:**
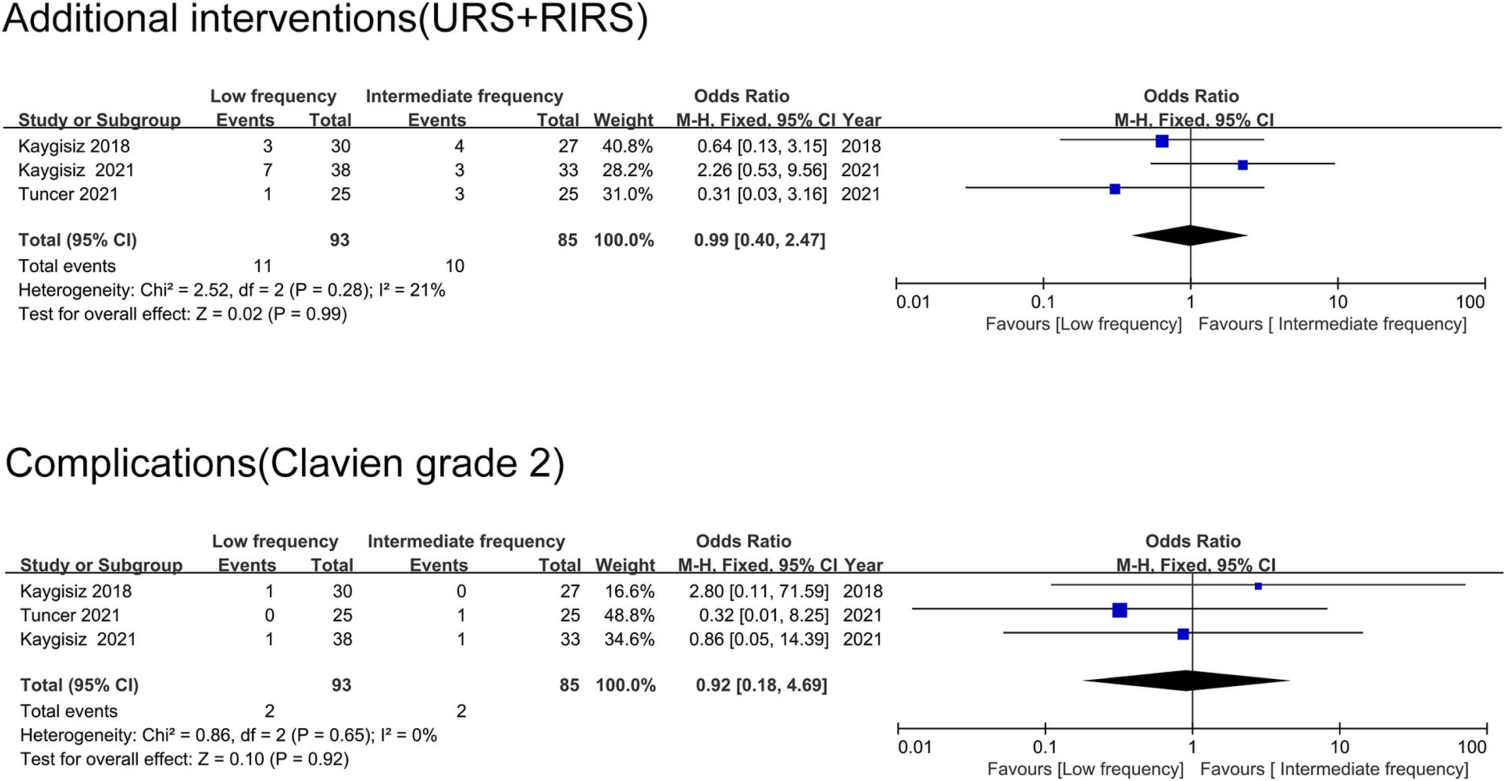
The pooled analyses of additional interventions after ESWL and major complications.

Additional interventions applied in the eligible studies were inconsistent, including percutaneous nephrolithotomy (PCNL), ureteroscopic laser lithotripsy (URS) and retrograde intrarenal surgery (RIRS). Additionally, to facilitate inclusion analyses, due to the characteristics of the interventions, we classified URS and RIRS as the same grade, while PCNL was classified as another grade. The pooled analyses of additional interventions with URS or RIRS were conducted between the low-frequency group and intermediate-frequency group, and no statistically significant difference was found (OR = 0.99 95%CI 0.40∼2.47, *p* = 0.99) (Figure 3). No study recorded PCNL as the additional intervention in the comparison between low frequency and intermediate frequency; thus, a meta-analysis could not be performed. Only one study compared URS or RIRS as additional interventions between intermediate-frequency SWL and high-frequency SWL. URS was performed in 3 cases in the intermediate-frequency group and 1 case in the high-frequency group. Two RCTs reported the application of PCNL as a method to enhance stone-free status after unsuccessful SWL management. Significantly more PCNL cases were recorded in the high-frequency group compared with the intermediate-frequency group.

### Major complications

Major postoperative complications involving fever, hematuria, pain and urinary tract infection were presented in terms of the modified Clavien classification across all the eligible studies, and no severe complications were recorded. In the comparison between the low-frequency group and intermediate-frequency group, two studies filed postoperative Clavien grade 1 complications, and the results showed superiority in the intermediate-frequency group (26%∼44% in the low-frequency group, 11%∼24% in the intermediate-frequency group). While three studies recorded postoperative Clavien grade 2 complications, and no significant difference was perceived between the groups (OR = 0.92 95%CI 0.18∼4.69, *p* = 0.92) (Figure 3). The difference was not statistically significant between the intermediate-frequency and high-frequency groups regarding the postoperative complication rates in the eligible studies (4%∼6.6% in the intermediate-frequency group, 4%∼13.3% in the high-frequency group).

### Efficiency quotient

The efficiency quotient was applied in all the involved studies and was calculated using the same formula: stone-free rate (%) × 100/[100 + retreatment rate (%) + rate of auxiliary procedures (%)]. The data were comparable between the low-frequency group (0.41∼0.58) and the intermediate frequency group (0.36∼0.64). However, a higher EQ was detected in the intermediate frequency group compared with high-frequency group (0.17∼0.34) in the eligible studies.

## Discussion

How to balance the increasing success rate with fewer complications remains a puzzling problem in the ESWL treatment of urolithiasis. So far, two nomograms have been conducted for predicting the stone-free status after ESWL in children with urolithiasis (18, 19). A previous history of ipsilateral stone treatment, age, gender, stone burden and location were found to be independent prognostic factors. A recently published study found that BMI might also be a significant parameter in the prediction of final success rates after ESWL (20). Onal et al. observed age as an important factor in the prediction of stone-free levels after ESWL. The younger age group (5 years old or younger) exhibited the highest risk of stone-free status, while the older age group (more than 10 years old) had the lowest possibility. Various studies focusing on urolithiasis in adults have demonstrated that the number of stones and the location (lower pole peculiarly) are crucial determinants for post-ESWL stone-free status (21, 22). Regarding the stone burden, the highest overall stone-free rate was observed in ESWL for stones smaller than 1 cm. As reported, a prior history of ipsilateral stone management may generate a negative impact on the stone-free rate of subsequent ESWL. Although the rationale for the finding remains unclear, prior stone management may induce scarring and influence the normal contraction as well as peristalsis of the ureter, leading to slight delays in urinary drainage. In the end, the excretion of stone fragments after ESWL is affected.

Nevertheless, the difference was not statistically significant with respect to the predictive characteristics among the different frequency groups in the current study. These findings further brought us the benefits of evaluating the reasonable effects of low, intermediate and high shock wave delivery rates during ESWL on SFR as well as postoperative complications in the pediatric population.

With increasing popularity, the percentage of pediatric patients treated with ESWL is also increasing, but despite interest, data related to the anesthetic management of these patients remain limited. ESWL may cause anxiety, fear, and pain in pediatric patients. In the present clinical practice, anesthesia is required by most pediatric patients to alleviate procedure-related pain as well as to avoid movements and reactions in the course of management. Recent literature has highlighted the potentially harmful effects of lengthy anesthetic exposure on brain development in pediatric patients (23).

In our analyses, no statistically significant difference was established regarding the total anesthesia time in the low frequency group and intermediate frequency group. However, due to the physical nature, more shockwaves and sessions are required for stone fragmentation in the intermediate frequency group than in the low frequency group. This may be the explanation for the similar median anesthesia time. One trial observed that pediatric patients treated with intermediate-frequency ESWL exhibited significantly longer anesthesia times than patients treated with high-frequency ESWL. The anesthesia time was similar between the intermediate frequency and high frequency groups in the other RCTs.

Treatment duration is a significant factor that may affect the application of different frequencies. In adults, a prominently longer treatment time was observed in the low frequency group than in the intermediate frequency or high frequency groups. Different from adults, longer treatment duration may influence patient tolerance in pediatric patients. On the other hand, the longer treatment duration, the dosage of agents and time of sedation or general anesthesia required may also increase. Only one trial recorded the total ESWL time among the eligible studies, and the results suggested that low frequency ESWL may result in longer treatment duration; however, the small sample size limits the possibility of performing the meta-analysis.

Outcomes of ESWL in the pediatric population vary by a large margin in stone-free rates and retreatment rates. The stone-free rate in children ranges from 59.2% to 94.8% (24–26), while the retreatment rates can reach 83%. However, these parameters may vary depending on the definition of success and the type of lithotripter. Abundant cavitation bubbles generated at the higher frequency may be the other explanation for the difference in success rates between the groups (27, 28). An acoustic mechanism proposed by Pischalnikov et al. suggested that a lower frequency ESWL is relatively favorable for stone fragmentation (29). Bubbles can be generated when the surface of the stone is exposed to the pressure of shock waves. The collapse of bubbles on the stone surface releases high-energy waves that eventually lead to the fragmentation of the stone. When the frequency of the ESWL increases, more cavitation bubbles can be generated on the stone surface, theoretically enhancing the efficiency of stone fragmentation. However, the remaining bubbles can neither dissipate before the arrival of the next shock wave nor continue to dissipate the stone but act as a barrier; thus, the energy transmitted by the shock wave is weakened and eventually reduces the efficacy. So far, two systematic reviews and meta-analyses have been performed to determine the optimal frequency of ESWL for urinary tract stone disease in adults (30). The pooled results were consistent. Low- and intermediate-frequency ESWL exhibited higher success rates than high-frequency ESWL, while no significant differences were found in the success rate between low-frequency ESWL and intermediate-frequency ESWL. Interestingly, Li et al. found that in the treatment of small stones (diameter less than 10 mm), the success rate showed no significant difference in the three groups; when the stones became larger, lower frequencies began to emerge with advantages in the success rates (31). Due to limitations on the data of original studies, whether high frequency has potential benefits in processing small stones in pediatrics could not be evaluated. On the whole, compared with adults, the efficacy of ESWL for the management of urinary stones in children is higher. This could be explained by the shorter and more elastic ureter in the pediatric population, which results in fewer fragment impaction and the easier transit of stones. In addition, children have better mobility than adults, which may also help stone passage.

With regard to the additional procedure required, Salem et al. found that compared with the intermediate-frequency group, significantly higher secondary procedure rates were observed in the high-frequency group (11). However, PCNL was the only additional intervention applied in Salem’s study; thus, the pooled result should be interpreted carefully. The total number of sessions applied also served as an indicator of success in the current analyses. The pooled results suggested a relatively low efficacy in high-frequency ESWL.

Although ESWL is considered a noninvasive procedure, it is not entirely safe. Renal exposure to SWs can lead to vascular rupture, resulting in parenchymal hemorrhage or subcapsular hematoma formation and can lead to serious complications (32). Postoperative complication rates of ESWL in children vary from 1.5% to 35%, depending on the definition of complication grades and the duration of follow-up. The most probable reported early complications are steinstrasse (no case recorded in the current study) and renal colic. As reported, steinstrasses occurred in up to 8.5% of participants, while the risk was higher for younger children or those with a larger stone load. Both *in vitro* and *in vivo* animal studies have proven that slow rates of shock wave delivery are associated with less tissue damage (33, 34). Kang et al. showed that low frequency ranked the highest for low complication rate, similar to adult patients (30), we also found low frequency exhibited higher Clavien grade 1 complications compared with intermediate frequency. Although most of these complications are mild, the results still suggest the potential advantage of intermediate frequency ESWL. Other anesthesia-related perioperative complications, e.g., atrial extrasystole, ventricular extrasystoles, and cardiac arrhythmia, were also recorded in the involved studies; nevertheless, the data were not sufficient to be included in the final meta-analyses. Accordingly, additional studies on the complications of ESWL in relation to the applied SW frequency are needed.

The current study has several limitations: 1. Stone composition could be an important parameter that may influence the stone-free rate. Salem et al. reported that calcium oxalate dihydrate is the main component in stone composition analysis in both intermediate frequency and high frequency groups (11). Nevertheless, stone composition is often unclear when deciding on treatment in clinical practice. 2. The impact of the total number of shock waves delivered was not assessed in the current study because the data were not available, which may introduce confounding bias. 3. As only two RCTs compared the efficacy and safety between high-frequency and intermediate-frequency groups, we only stated the results without performing pooled analyses. Also, such a small number of included trials and sample sizes were unable to make strong conclusions. Thus, well-designed and high-quality multi-center long-term RCTs with large sample sizes are required to validate the findings. 4. Different matching criteria, as well as the assessment of outcomes, were applied in the eligible studies. These differences may lead to interstudy heterogeneity. 5. The type of lithotripter machine is known to influence SFR in adults, and the data were sparse in the pediatric population; thus, a comparison could be performed.

## Conclusions

The data acquired from the pooled analyses revealed that ESWL frequency may affect anesthesia time, overall success rate and possible procedure-related complications; thus, a precise balance needs to be achieved on the basis of these parameters. Slowing frequency from high frequency to intermediate or low frequency increased the overall success rates of ESWL for pediatric patients with upper urinary stones. Low to intermediate seemed to be an optimal frequency. Nevertheless, future large-volume, well-designed RCTs are awaited to confirm and update the findings of this analysis.

## Data Availability

The original contributions presented in the study are included in the article/Supplementary Material, further inquiries can be directed to the corresponding author/s.
